# RESILIENCE IN DIVERSE SOLO OLDER GRANDMOTHERS RAISING ADOLESCENT GRANDCHILDREN

**DOI:** 10.1093/geroni/igae098.2422

**Published:** 2024-12-31

**Authors:** Tina Peterson

**Affiliations:** University of Cincinnati, Cincinnati, Ohio, United States

## Abstract

Grandfamilies scholars often focus on shared caregiving responsibilities supported by a spouse or partner when parenting younger grandchildren. Less attention has been given to solo older grandparents raising adolescent grandchildren. This research will apply Dannison and colleagues’ (2011) framework of the 5 P’s (proud, positive, persistent, protective, problem-solvers) of Resilient Grandparent Caregivers to explore resiliency in solo older grandparents raising adolescent grandchildren. Solo grandparent caregivers (n=8) participated in a phenomenological study to examine Caregiving in Later Life by Grandparents Raising Adolescent Grandchildren. Participants were primary caregiver for a grandchild 12 or older; grandchild in home at least 3 days weekly; and grandparent 40 or older. Data collection via semi-structured interviews occurred across three states between March 2015 and June 2015. Interviews were audiotaped, transcribed verbatim, and analyzed using conventional content analysis. Racially and ethnically diverse grandmothers (100%) reported an average age of 61 years (range 56 to 67) with caregiving responsibility for grandchildren with a median age of 14 years. These solo older grandmothers described being proud (e.g. joy, energy, grandchildren’s talent, liveliness, etc.); positive (e.g. telling jokes, picking battles, reframing behavior, music, etc.); persistent (e.g. kinship foster care placement, most challenging role, navigating multiple responsibilities, etc.) protective (e.g. setting boundaries, honoring holiday celebrations, structure, etc.) and problem-solvers (e.g. seeking therapy, advocating within schools, generational gifts, resolving purchasing for multiple kids, instilling time management, etc.). Reflections on resilience from diverse solo older grandmothers raising adolescent grandchildren can promote strategies to support and advocate for underserved grandfamilies.

